# High-resolution surface electromyographic activities of facial muscles during mimic movements in healthy adults: A prospective observational study

**DOI:** 10.3389/fnhum.2022.1029415

**Published:** 2022-12-12

**Authors:** Nadiya Mueller, Vanessa Trentzsch, Roland Grassme, Orlando Guntinas-Lichius, Gerd Fabian Volk, Christoph Anders

**Affiliations:** ^1^Division Motor Research, Pathophysiology and Biomechanics, Department of Trauma, Hand and Reconstructive Surgery, Jena University Hospital, Friedrich-Schiller-University Jena, Jena, Germany; ^2^Department of Otolaryngology, Jena University Hospital, Friedrich-Schiller-University Jena, Jena, Germany; ^3^Department of Prevention, Biomechanics, German Social Accident Insurance Institution for the Foodstuffs and Catering Industry, Erfurt, Germany; ^4^Facial-Nerve-Center Jena, Jena University Hospital, Jena, Germany; ^5^Center for Rare Diseases, Jena University Hospital, Jena, Germany

**Keywords:** facial muscle activations, facial movement analysis, facial nerve (FN), surface EMG (sEMG), high-density sEMG, face

## Abstract

**Objectives:**

Surface electromyography (sEMG) is a standard tool in clinical routine and clinical or psychosocial experiments also including speech research and orthodontics to measure the activity of selected facial muscles to objectify facial movements during specific facial exercises or experiments with emotional expressions. Such muscle-specific approaches neglect that facial muscles act more as an interconnected network than as single facial muscles for specific movements. What is missing is an optimal sEMG setting allowing a synchronous measurement of the activity of all facial muscles as a whole.

**Methods:**

A total of 36 healthy adult participants (53% women, 18–67 years) were included. Electromyograms were recorded from both sides of the face using an arrangement of electrodes oriented by the underlying topography of the facial muscles (Fridlund scheme) and simultaneously by a geometric and symmetrical arrangement on the face (Kuramoto scheme). The participants performed a standard set of different facial movement tasks. Linear mixed-effects models and adjustment for multiple comparisons were used to evaluate differences between the facial movement tasks, separately for both applied schemes. Data analysis utilized sEMG amplitudes and also their maximum-normalized values to account for amplitude differences between the different facial movements.

**Results:**

Surface electromyography activation characteristics showed systematic regional distribution patterns of facial muscle activation for both schemes with very low interindividual variability. The statistical significance to discriminate between the different sEMG patterns was good for both schemes (significant comparisons for sEMG amplitudes: 87.3%, both schemes, normalized values: 90.9%, Fridlund scheme, 94.5% Kuramoto scheme), but the Kuramoto scheme performed considerably superior.

**Conclusion:**

Facial movement tasks evoke specific patterns in the complex network of facial muscles rather than activating single muscles. A geometric and symmetrical sEMG recording from the entire face seems to allow more specific detection of facial muscle activity patterns during facial movement tasks. Such sEMG patterns should be explored in more clinical and psychological experiments in the future.

## Introduction

Facial muscle contractions form mimic expressions important for many functions in human behavior (Cattaneo and Pavesi, 2014). They enable facial motor control, for instance, for eye closure or mouth control during drinking and eating. Moreover, facial expressions reflect emotions and are essential for non-verbal communication. Therefore, facial electromyography (EMG) represents a standard in psychological experiments for psychophysiological measures to test the group activity of some facial muscles and its association with specific emotions (Hubert and de Jong-Meyer, 1991). Furthermore, EMG investigations of facial muscles are a standard for facial muscle diagnostics in patients with facial nerve and muscle diseases (Guntinas-Lichius et al., 2020). Facial EMG recordings in a psychological and clinical setting usually use only two to six pairs of needle or surface electrodes to record the resting state or voluntary movements of some facial muscles on the assumption that this reflects the respective mimic muscles as a whole. This approach and assumption neglect that the facial muscular system forms a complex interdependent system of the individual anatomically defined facial muscles that are connected to the skin (Cattaneo and Pavesi, 2014). They have no fixed bone-based insertion points, are interwoven, and are partly overlapping. The activation of one muscle with the aim to induce a movement in a certain direction involuntarily elicits the activation of other facial muscles. This activation of other facial muscles is needed to create a fixation point for the intended movement. Therefore, each facial exercise leads to a complex activation of almost all facial muscles (Schumann et al., 2021).

To approach a realistic setting, Fridlund and Cacioppo recommended surface EMG (sEMG) recordings of 1 masticatory and 10 facial muscles (Fridlund and Cacioppo, 1986) for psychophysiological investigations. Kuramoto et al. (2019) recommended the use of 21 sEMG electrodes in an EEG-like arrangement. The idea behind this particular approach is that such a muscle site-independent recording over the face might better reflect the complex facial activity during exercises or emotional expressions (Kuramoto et al., 2019). Recently, experimental high-density sEMG (HD sEMG) applications with 90 electrodes even allowed the visualization of mimic muscle activation projected on the facial surface (Cui et al., 2020). Lately, we published a study and an atlas on facial muscle activation patterns based on multichannel sEMG (Schumann et al., 2010, 2021). The sEMG amplitude characteristics were transposed into a color-coded atlas of voluntary facial muscle activation tasks. We could show that facial muscles act more as a whole connected muscle network than as single facial muscles, responsible for certain movements.

As a next step and in a new clinical trial, we, therefore, wanted to use both the sEMG electrode schemes of Fridlund and Cacioppo (1986) and Kuramoto et al. (2019) synchronously to analyze which scheme allows optimal visualization of the facial muscle activity of the entire face during specific facial movements. Furthermore, we asked which of the two schemes is better suited to distinguish different facial expressions from each other.

## Materials and methods

### Healthy participants

The study included 36 healthy adult volunteers (52.8% women) with no neurological diseases or history of facial surgery (mean age: 33.3 ± 15.2 years; range: 18–67). All participants gave written informed consent to participate in this study. The ethics committee of the Jena University Hospital approved this study (No. 2019-1539). The individual shown in the figures of this manuscript has given written informed consent to publish these case details.

### Standardization of mimic exercises

**FIGURE 1:**
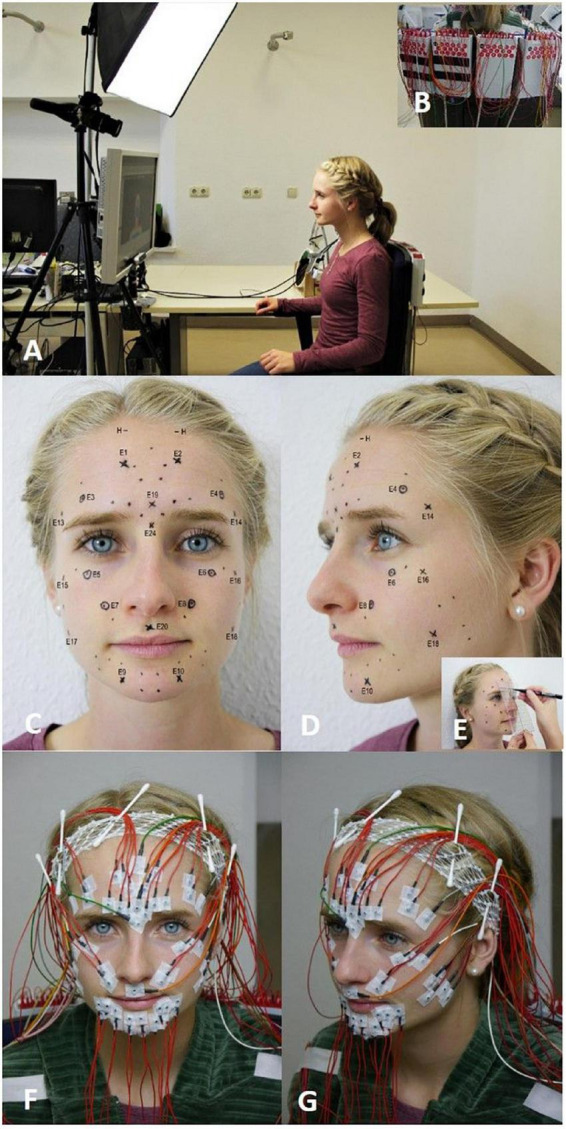
The experimental setup. **(A)** The participant is sitting at a standardized distance from the computer screen and is following the instructions. **(B)** The electromyography (EMG) amplifiers are placed in the back of the chair. **(C–E)** EMG electrode schemes after Fridlund and Cacioppo (dots) and after (Kuramoto et al., 2019) (crosses) are marked in the face of the participant with the help of a standardized template (circled crosses refer to electrodes shared by both schemes). **(F)** Frontal view on the fixed surface EMG (sEMG) electrodes. **(G)** Side view on the fixed sEMG electrodes.

The participants were instructed about the sequence of the examination and the facial exercises. The experimental workup is shown in Figures 1A,B. Room temperature and relative humidity were controlled to be approximately 24°C and 40–65%, respectively. Participants sat in a relaxed upright position in front of a computer screen and followed a self-explanatory video tutorial published recently (Schaede et al., 2017). This provides standardized and reliable instruction to perform facial movements guided by a human instructor (Volk et al., 2019). Participants followed the instructions on the computer screen. Details are presented elsewhere (Volk et al., 2019). The sequence of the facial movements is shown in Supplementary Table 1.

### Facial sEMG registration

**FIGURE 2:**
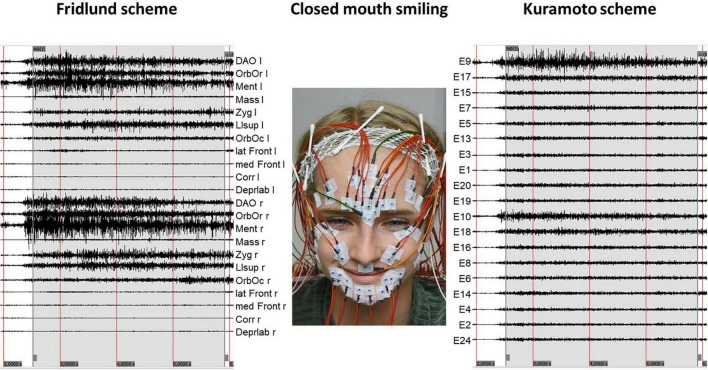
Example of an sEMG recording during a specific task (here closed mouth smiling). The recordings of Fridlund electrodes are shown on the **(left side)**. The recording related to the Kuramoto electrodes is shown on the **(right side)**. The numbering of the electrodes of the Kuramoto scheme is following the original numbering of Kuramoto et al. (2019). The positioning of the electrodes is following the muscle anatomy. l, left side; r, right side; DAO, depressor anguli oris muscle; OrbOr, orbicularis oris muscle; Ment, mentalis muscle; Mass, masseter muscle; Zyg, zygomatic muscle; Llsup, levator labii superioris muscle; OrbOc, orbicularis oculi muscle; lat Front, lateral part of the frontalis muscle; med Front, medial part of the frontalis muscle; Corr, corrugator supercilii muscle; Deprlab, depressor labii inf.

Surface electromyography measurement was applied using a monopolar montage utilizing reusable surface electrodes (Ag-AgCl discs, diameter of 4 mm, DESS052606, GVB-geliMED, Bad Segeberg Germany). The reference electrodes were positioned at both mastoid processes and connected. The sEMG recording was performed with a multichannel EMG system (gain: 100, frequency range: 10–1,861 Hz; sampling rate: 4,096/s; resolution: 5.96 nV/bit; DeMeTec, Langgöns, Germany). Electromyograms were recorded from both sides of the face simultaneously using both the arrangement of electrodes by Fridlund and Cacioppo (1986) and by Kuramoto et al. (2019) (cf. Figures 1C–G). The schemes were labeled as “Fridlund” and “Kuramoto.” The Fridlund scheme is following the topography of the facial muscles. Eleven pairs of electrodes (interelectrode distance for each pair: 1 cm) per hemiface were needed. The corrugator supercilii and depressor supercilii muscles shared one electrode simultaneously. Hence, 21 electrodes were placed on each side of the face. In total, 42 electrodes were needed for the Fridlund scheme. In contrast, the Kuramoto scheme does not take muscle topography into consideration. Similar to an electroencephalogram (EEG), the electrodes were placed in symmetrical standard positions with fixed distances between the electrodes (Beniczky and Schomer, 2020). Eight active electrodes were placed on each side of the face. In addition, three electrodes were placed in the midline of the face. Hence, 19 electrodes were needed in total for the Kuramoto scheme. Six electrodes were shared by the simultaneous application of both schemes (cf. Figures 1C–G). One ground electrode and one reference electrode on each mastoid were needed. Therefore, overall 58 electrodes were placed on the face for both the Fridlund and the Kuramoto scheme and the additional electrodes. An example of an EMG recording for one task is shown in Figure 2.

To account for possible technical and also physiological artifacts prior to any further analysis, signals were centered and bandpass filtered between 10 and 500 Hz. Also, a 50 Hz notch filter was applied to account for interferences from the electrical circuit.

For the Fridlund scheme, from the monopolar measured electrodes, bipolar channels were calculated by subtracting the signals from the respective electrode pairs (Fridlund and Cacioppo, 1986). Data for the Kuramoto scheme were monopolar analyzed. sEMG amplitudes were quantified as mean RMS values (single interval duration for calculation: 125 ms) during the steady state contraction phases of every movement and sEMG channel. Also, maximum-normalized data were calculated to account for individual and movement-dependent amplitude variations and also general amplitude differences between the facial movements.

### Heat maps for visualization of the sEMG activity

Heat maps were calculated to provide a topographic impression of the myogenic activity distribution over the face for both schemes. A modified 4-nearest neighbor interpolation of the EMG RMS values was used similar to the EEG mapping with the inverse square of the distance as weight (Jäger et al., 2016). Interpolation in EEG mapping is often performed on the basis of spherical functions because the conducting area in EEG is approximately a hemisphere (Lehmann, 1992). This precondition is not given in the face. Therefore, the 4-nearest neighbor interpolation was used. To avoid spatial discontinuity, the weight of the most distant (fourth) electrode was steadily pulled to zero in the vicinity of the change between two fourth neighbors (Soong et al., 1993).

### Statistics

Separately for each scheme, we applied a linear mixed-effects model (LMM) to evaluate the main effects of the parameters “movement,” “side,” and “electrode position,” together with their interactions. “Movement,” “side,” and “electrode position” were modeled as fixed effects with a random intercept per subject. Initially, all main effects and interactions were calculated, but for the final analysis, only the significant main effects together with significant interactions remained in the calculation. Adjustment for multiple comparisons for differences between the tested facial movements was performed by the least significant difference. To differentiate between amplitude-driven and distribution-driven differences, these calculations were applied separately for the RMS and the normalized dataset. The significance level was set to 5%.

## Results

### Effects of the specific movement, electrode position, and side of the face

Independent of the analyzed scheme and dataset, the initial statistical calculation revealed significant main effects of the parameter “movement” (RMS dataset, Fridlund: *p* < 0.001, *F*: 290.5; Kuramoto: *p* < 0.001, *F*: 364.9; normalized dataset, Fridlund: *p* < 0.001, *F*: 96.3; Kuramoto: *p* < 0.001, *F*: 384.3) and “electrode position” (RMS dataset, Fridlund: *p* < 0.001, *F*: 309.1; Kuramoto: *p* < 0.001, *F*: 220.7; normalized dataset, Fridlund: *p* < 0.001, *F*: 370.3; Kuramoto: *p* < 0.001, *F*: 230.8), but not for “side” in the Fridlund scheme. In the Kuramoto scheme, the parameter “side” had a significant effect in the RMS dataset (*p* = 0.027, *F*: 4.9). The pairwise comparisons revealed significant differences between both facial sides and the centrally located electrodes (left and right vs. central *p* < 0.001). The values were always lower for the centrally located electrodes. There was no difference between the left and right sides (*p* = 0.984). Hence, the left and right body sides themselves had no significant main effect. Therefore, we decided to further analyze the RMS dataset of the Kuramoto scheme independent of the body side.

Interactions were significant for the parameters “movement*electrode position” (RMS dataset, Fridlund: *p* < 0.001, *F*: 87.4; Kuramoto: *p* < 0.001, *F*: 77.9; normalized dataset, Fridlund: *p* < 0.001, *F*: 104.3; Kuramoto: *p* < 0.001, *F*: 78.8) and “side*electrode position” (RMS dataset, Fridlund: *p* = 0.003, *F*: 2.5; Kuramoto: *p* < 0.001, *F*: 17.2; normalized dataset, Fridlund: *p* < 0.001, *F*: 16.6; Kuramoto: *p* < 0.001, *F*: 17.2). In the Kuramoto scheme also, the interaction “movement*side” was a significant parameter (*p* = 0.003, F: 2.7). Based on these results, the different facial movements were then compared with each other.

### sEMG recordings with the Fridlund scheme

**FIGURE 3:**
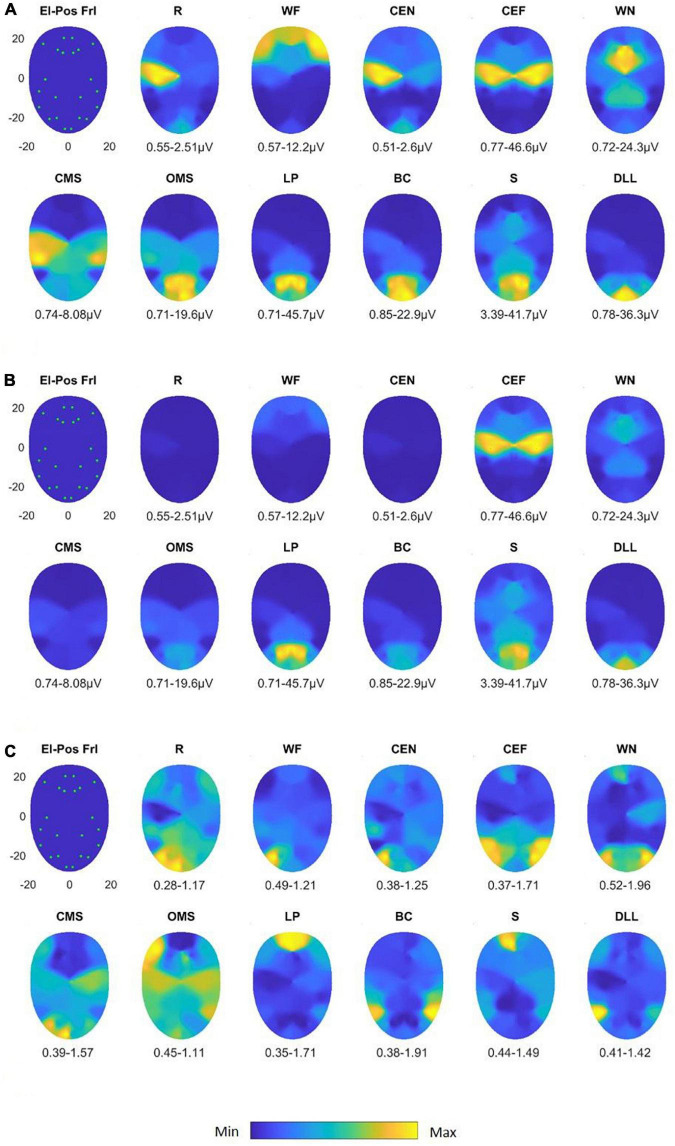
Activation heat maps of facial muscle activity during different mimic exercises using the Fridlund scheme (El-Pos Fri). According to the provided color bars, blue stands for low values and yellow for high values. Below each map, the respective minimal (Min) and maximal (Max) values are provided. **(A)** Heat maps with always individual Min to Max scaling; **(B)** heat maps scaled according to the Min and Max values across all facial movements. **(C)** Variability (variation coefficient) heat maps. R, at rest; WF, wrinkling of the forehead; CEF, closing the eyes normally; CEF, closing the eyes forcefully; WN, wrinkling of the nose; CMS, closed mouth smiling; OMS, open mouth smiling; LP, lip puckering; BC, blowing-out the cheeks; S, snarling; DLL, depressing lower lip.

**FIGURE 4:**
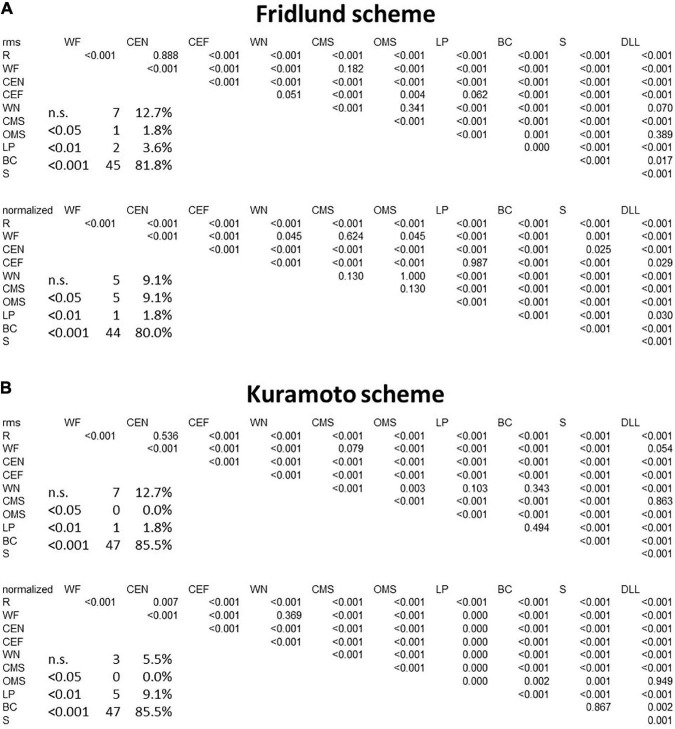
Results of the between-movement comparisons by applying a linear mixed-effects model. **(A)** Fridlund scheme. **(B)** Kuramoto scheme. Upper row for both schemes: row data; lower row: normalized data. Significance levels are color-coded: *p* < 0.05: pink, *p* < 0.001: orange, *p* < 0.001: red. Also, the absolute numbers and relative numbers of non-significant and significant differences are provided. R, at rest; WF, wrinkling of the forehead; CEF, closing the eyes normally; CEF, closing the eyes forcefully; WN, wrinkling of the nose; CMS, closed mouth smiling; OMS, open mouth smiling; LP, lip puckering; BC, blowing-out the cheeks; S, snarling; DLL, depressing lower lip; n.s., not significant.

The heat map presentation of the 11 facial movements is shown in Figures 3A,B. Activation characteristics showed systematic regional distribution patterns as could be expected by the facial movements. The variability of these values across the face is provided in an amplitude-normalized fashion, i.e., as variation coefficients (VCs), and are presented in Figure 3C. Some facial tasks showed a very low interindividual variability of the activation patterns (for instance, “Wrinkling the forehead”), whereas others showed a higher variability (for instance, “Smiling with open mouth”). Overall, the variability of the activation in μV was very low. In Figure 4A, the results of the LMM calculations of the pairwise comparisons between all facial movements are provided for the RMS and normalized datasets. The sEMG recording allowed highly significant discrimination between most of the exercises (*p* < 0.0001) but some failed. The comparisons of the tasks “depress lower lips” vs. “wrinkle nose” and “open mouth smile,” “close eyes forcefully” vs. “wrinkle nose” and “lip pucker,” “wrinkle forehead” vs. “closed mouth smile,” “lip pucker” vs. “close eyes forcefully,” and “rest” vs. “close eyes normally” showed no significant difference (all *p* > 0.05). After normalization, the number of non-significant comparisons dropped, but still the comparisons “closed mouth smile” vs. “open mouth smile,” “wrinkle forehead,” and “wrinkle nose” and also “close eyes forcefully” vs. “lip pucker” remained non-significant (all *p* > 0.05).

### sEMG recording with the Kuramoto scheme

**FIGURE 5:**
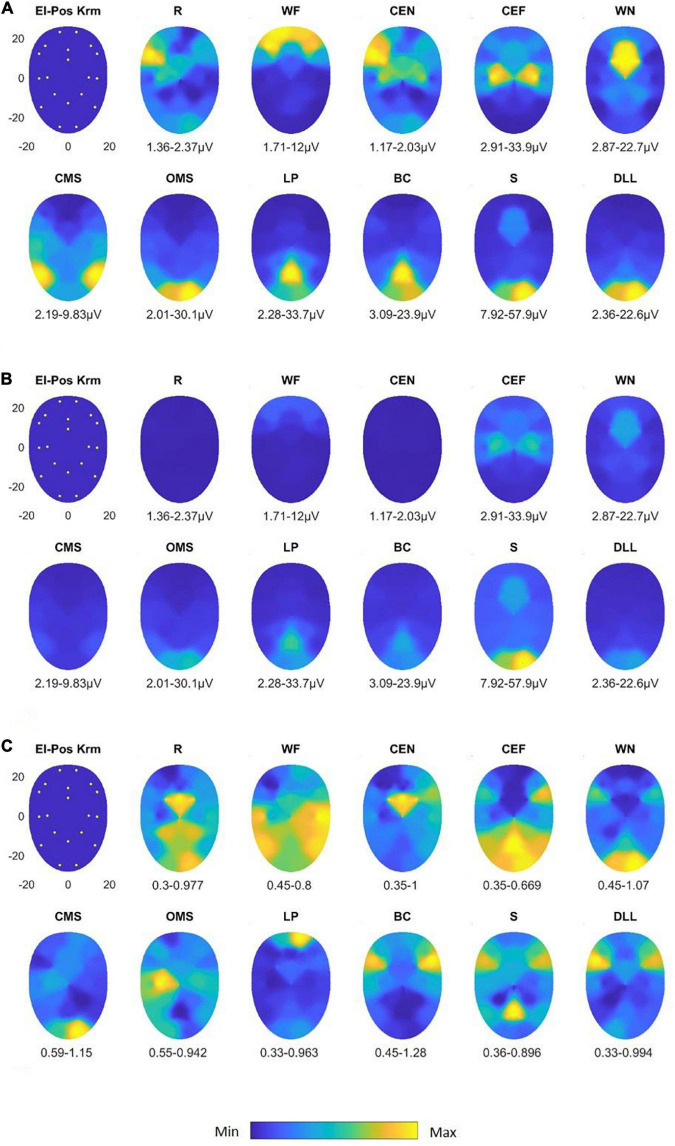
Activation heat maps of facial muscle activity during different mimic exercises using the Kuramoto scheme (El-Pos Krm). According to the provided color bars, blue stands for low values and yellow for high values. Below each map, the respective minimal (Min) and maximal (Max) are provided. **(A)** Heat maps with always individual Min to Max scaling; **(B)** heat maps scaled according to the Min and Max values across all facial movements. **(C)** Variability (variation coefficient) heat maps. R, at rest; WF, wrinkling of the forehead; CEF, closing the eyes normally; CEF, closing the eyes forcefully; WN, wrinkling of the nose; CMS, closed mouth smiling; OMS, open mouth smiling; LP, lip puckering; BC, blowing-out the cheeks; S, snarling; DLL, depressing lower lip.

In Figures 5A,B, the maps for the Kuramoto scheme of all 11 facial movements and in Figure 5C, the respective VC values are provided. As can be seen from Figure 4B, the number of highly significant differences between separate and specific facial movements was considerably larger as could be obtained by the application of the Fridlund scheme (cf. Figure 4A). The following comparisons of facial movement tasks could not reach significant results: “wrinkle nose” vs. “lip pucker” and “blow-out cheek,” “depress lower lip” vs. “wrinkle forehead” and “closed mouth smile,” “wrinkle forehead” vs. “closed mouth smile,” and finally “rest” vs. “close eyes normally.”

After normalization of the data, nearly all facial exercises revealed highly specific sEMG activity patterns (*p* < 0.0001). Only “wrinkle nose” vs. “wrinkle forehead,” “blow-out cheek” vs. “snarl,” and “depress lower lip” vs. “open mouth smile” failed to reach the required significance level (all *p* > 0.05).

## Discussion

The present study was the first prospective clinical trial directly and synchronously comparing the facial sEMG electrode arrangements of Fridlund and Cacioppo vs. the new arrangement of Kuramoto et al. in the same group of healthy probands (Fridlund and Cacioppo, 1986; Kuramoto et al., 2019). Overall, both sEMG schemes allowed to record of characteristic and discriminable facial muscle activation patterns during different facial movement tasks. The geometric sEMG recording from the entire face introduced by Kuramoto et al. seemed to allow more specific detection of facial muscle activity patterns during facial movement tasks (Kuramoto et al., 2019). As shown by others, there was no relevant side difference (Schumann et al., 2010, 2021; Kuramoto et al., 2019; Cui et al., 2020). In contrast, HD sEMG recordings of the facial and neck muscles show mainly a symmetrical activation but also some side differences during phonation tasks (Zhu et al., 2022). Hence, it might be that the degree of left/right symmetry might be task-dependent.

Since its publication in 1986, the recommendations of Fridlund and Cacioppo for sEMG electrode placements for a differential recording over the most important facial muscles [Figure 1 in Fridlund and Cacioppo (1986)] probably become the most popular facial recording scheme. More than 1,200 articles (Web of Science, August 2022, mainly psychological research) refer to this scheme when describing their facial sEMG approach. It is important to notice that the Fridlund scheme covers nine facial muscles (called by Fridlund and Cacioppo as “major” facial muscles) and not all facial muscles. Second, many articles cite the Fridlund scheme, even when the recordings are just performed from some of the nine facial muscles. As shown in the present study, the Fridlund scheme is a powerful tool when placing all 22 electrode pairs. We have doubts, and that was not the aim of Fridlund and Cacioppo, that a reduced scheme does allow to control of specific facial muscle activations during mimetic investigations. The accuracy of an EMG activation pattern to reflect a specific mimetic task is not only important for clinical or psychosocial studies. There are first approaches to use specific human facial gestures based on EMG data for human–machine interface (HMI) technologies for steering of machines (Hamedi et al., 2011). This will only work when reliable sEMG facial muscle patterns are used.

In this light, it is important to bring to mind that although facial muscles are conventionally classified as individual muscles, these muscles are interconnected, and movements are interdependent on each other (Cattaneo and Pavesi, 2014). Even more, the activation of one muscle may define the pulling direction of another facial muscle just in this actual moment. Therefore, most facial and emotional expressions need the coordinated action of several facial muscles (cf. Figure 2: not only muscles around the mouth are activated for smiling). Furthermore, the correct identification of all respective facial muscles requires highly trained personnel and is time-consuming to produce reliable results. Also, the post-processing (monopolar recording has to be converted into bipolar channels) bears some risks. For this reason, the Kuramoto scheme is of such interest. For this approach, called by the authors “myogenic potential topography,” the electrodes are placed on the face with fixed inter-electrode distance covering the entire face without reference to the underlying facial muscles. In the original study, the probands had to perform two mimic expressions (smiling and a disgusting face) different from the present study. Therefore, a comparison of the results is not possible. Using much more movement tasks, we were able to show that the Kuramoto scheme allows discrimination of the most important (non-emotional) facial movements. Cui et al. used six facial movements (raising eyebrows, closing eyes, bulging cheek, grinning, pouting, and wrinkling the nose) in combination with a facial high-density sEMG (HD sEMG) to produce center of gravity color-coded activation maps but finally refer the activation to single facial muscles (Cui et al., 2020).

The present study has some limitations. We analyzed only facial movement tasks. In the next experiments, we would like to compare both schemes when the probands perform emotional expressions. Furthermore, the differences between the reliability of the individual activation patterns for both sEMG schemes are unknown. In the present study, there was some interindividual variability in the muscle activation patterns. Facial muscle size shows some interindividual variability (Volk et al., 2014). This, but also age and gender, might influence activity patterns. The influence of these factors should be studied in larger cohorts of healthy adults. Furthermore, the performance of the tasks might vary if performed repeatedly on different days (Hess et al., 2017). Therefore, the retest reliability has to be investigated in future studies. Cui et al. used it for their HD sEMG work-up of 90 electrodes (Cui et al., 2020). In contrast to the presented schemes, it remains doubtful if such an elaborate experimental setup is applicable to clinical routine. It might be more interesting to develop easy-to-apply wearable, multichannel sEMG electrodes (Inzelberg et al., 2018, 2020; Gat et al., 2022). This would simplify routine application and would further allow investigations with freely moving probands, hence, would allow a more realistic setting, especially for psychosocial experiments.

## Conclusion

The high-resolution sEMG recording of healthy probands shows that specific facial movement tasks lead to complex facial muscle activation patterns. These patterns can only be detected if the entire facial muscles on both sides of the face are recorded simultaneously. Specific facial movement tasks lead to specific sEMG activation patterns. The discrimination seems to be more sensitive if an sEMG recording scheme with the geometrical arrangement (as typical for EEG) and independent of any facial muscle topography is used. Nevertheless, a classical sEMG recording scheme that is orientated to the topography of facial muscles produces also specific sEMG activation patterns for most facial movement tasks.

## Data availability statement

The original contributions presented in this study are included in the article/Supplementary material, further inquiries can be directed to the corresponding author.

## Ethics statement

The studies involving human participants were reviewed and approved by the Ethics Committee of the Jena University Hospital. The patients/participants provided their written informed consent to participate in this study. Written informed consent was obtained from the individual for the publication of any potentially identifiable images or data included in this article.

## Author contributions

OG-L, GV, and CA: conceptualization and supervision. OG-L and CA: first draft preparation. NM and VT: data acquisition. NM, VT, RG, and CA: data analysis. All authors contributed to the article and approved the final version.
