# Supplementary material for: High-resolution surface electromyographic activities of facial muscles during mimic movements in healthy adults: A prospective observational study
Source: Front Hum Neurosci. 2022 Dec 12;16:1029415. doi: 10.3389/fnhum.2022.1029415 (PMC9790991; doi:10.3389/fnhum.2022.1029415)
Supplement: Supplementary file 1 [file Table_1.pdf]

High-resolution surface electromyographic activities of facial muscles during mimic movements in healthy adults: A prospective observational study

Supplement Table 1

**Supplement Table 1.** EMG setting and facial exercises in standard sequence	
**No.**	**Step**
1	Marking of the two EMG schemes into the face
2	Placement of the electrodes
3	Sitting in front of the computer screen
4	Recording of the entire procedure
5	Starting of the self-tutorial and synchronous EMG recording
6	Introduction shown on the computer screen; all exercises are performed three times
7	Face at rest
8	Wrinkling of the forehead
9	Closing the eyes normally (Gentle eye closure)
10	Closing the eyes forcefully (Tight eye closure)
11	Nose wrinkling
12	Smiling with closed mouth
13	Smiling with open mouth
14	Lip puckering (Pursing lips)
15	Blowing-out the cheeks (Cheek blowing)
16	Snarling
17	Depressing lower lip
